# Magnesium Status and Calcium/Magnesium Ratios in a Series of Cystic Fibrosis Patients

**DOI:** 10.3390/nu14091793

**Published:** 2022-04-25

**Authors:** Marlene Fabiola Escobedo-Monge, Enrique Barrado, Joaquín Parodi-Román, María Antonieta Escobedo-Monge, Marianela Marcos-Temprano, José Manuel Marugán-Miguelsanz

**Affiliations:** 1 Department of Pediatrics of the Faculty of Medicine, Valladolid University, Avenida Ramón y Cajal, 7, 47005 Valladolid, Spain; jmmarugan@telefonica.net; 2 Department of Analytical Chemistry, Science Faculty, Campus Miguel Delibes, University of Valladolid, Calle Paseo de Belén, 7, 47011 Valladolid, Spain; ebarrado@qa.uva.es; 3 Science Faculty, Cadiz University, Paseo de Carlos III, 28, 11003 Cadiz, Spain; joaquin_parodi@yahoo.es; 4 Department of Chemistry, Science Faculty, University of Burgos, Plaza Misael Bañuelos sn, 09001 Burgos, Spain; antoitalia777@gmail.com; 5 Pediatric Service, University Clinical Hospital of Valladolid, Avenida Ramón y Cajal, 3, 47005 Valladolid, Spain; marianela_mt6@hotmail.com; 6 Section of Gastroenterology and Pediatric Nutrition, University Clinical Hospital of Valladolid, Avenida Ramón y Cajal, 3, 47003 Valladolid, Spain

**Keywords:** hypomagnesemia, hypermagnesemia, subclinical Mg deficiency

## Abstract

Magnesium (Mg) is an essential micronutrient that participates in various enzymatic reactions that regulate vital biological functions. The main aim was to assess the Mg status and its association with nutritional indicators in seventeen cystic fibrosis (CF) patients. The serum Mg and calcium (Ca) levels were determined using standardized methods and the dietary Mg intake by prospective 72 h dietary surveys. The mean serum Ca (2.45 mmol/L) and Mg (0.82 mmol/L) had normal levels, and the mean dietary intake of the Ca (127% DRI: Dietary Reference Intake) and Mg (125% DRI) were high. No patients had an abnormal serum Ca. A total of 47% of the subjects had hypomagnesemia and 12% insufficient Mg consumption. One patient had a serum Mg deficiency and inadequate Mg intake. A total of 47 and 82% of our series had a high serum Ca/Mg ratio of >4.70 (mean 4.89) and a low Ca/Mg intake ratio of <1.70 (mean 1.10), respectively. The likelihood of a high Ca/Mg ratio was 49 times higher in patients with a serum Mg deficiency than in normal serum Mg patients. Both Ca/Mg ratios were associated with the risk of developing cardiovascular disease (CVD), type 2 diabetes (T2D), metabolic syndrome (MetS), and even several cancers. Therefore, 53% of the CF patients were at high risk of a Mg deficiency and developing other chronic diseases.

## 1. Introduction

Cystic fibrosis (CF) is the most common autosomal recessive life-shortening multisystem disorder worldwide [1]. It is due to mutations in a single gene located on chromosome 7, the CFTR (Cystic Fibrosis Transmembrane Conductance Regulator) [2], which is involved in the regulation of the fluid volume in epithelial cells [3]. The CFTR gene encodes the CFTR protein that functions as a Cl-selective anion channel gated by cycles of ATP binding and hydrolysis at its nucleotide-binding domain [4]. It behaves as a chloride channel and the mutations cause an epithelial ion transport defect in the sweat glands and the respiratory, hepatobiliary, gastrointestinal (pancreas), and reproductive tracts [5]. The morbidity and mortality of this disease are mainly due to lung disease caused by infections and airway inflammation. Other clinical manifestations of this disease are stunted growth, pancreatic insufficiency, meconium ileus, and infertility [6]. 

An adequate nutritional status is an essential factor that reduces disease progression and improves survival [7]. Its goal in CF care includes achieving an optimal nutritional status to support growth and pubertal development in children and maintaining an optimal nutritional status in adult life [8]. One of the most important risk factors for nutritional deficiencies in CF is attributable to a decreased nutrient intake, especially during periods of acute illness [7] and growth. CF patients may have higher than normal requirements for salt, calcium (Ca), iron, zinc, and selenium due to the increased sweating, intestinal malabsorption, and chronic inflammation that are common in this chronic disease [9]. However, according to Gupta (2007), a deficient Mg status in CF patients is underrecognized, and the accurate incidence is unknown [10]. 

Magnesium (Mg) is an essential electrolyte for living organisms [11], having great influence on muscular contraction, blood pressure, insulin metabolism, cardiac excitability, vasomotor tone, nerve transmission, and neuromuscular conduction [12]. A chronic latent Mg deficiency (CLMD), which is often missed when only the serum Mg is analyzed, is already associated with an increased risk of disease [13]. Chronic Mg deficits (low intake and serum concentration) increase the production of free radicals implicated in the development of several chronic age-related disorders [14], including cardiovascular diseases (CVD), hypertension [15] and stroke [16], cardiometabolic syndrome, coronary hearth or artery disease (CHD or CAD) [17], sudden cardiac death, metabolic syndrome (MetS) [18], type 2 diabetes mellitus (T2D) [19], constrictive airway syndromes and asthma [20], depression, stress-related conditions and psychiatric disorders, Alzheimer’s disease (AD) and other dementia syndromes, muscular diseases (muscle pain, chronic fatigue, and fibromyalgia), bone fragility [14], chronic kidney disease (CKD) [21], neurodegenerative diseases, sarcopenia, frailty [20], and even cancer [14,20] (colorectal) [22]. 

Apart from the essential relationship with bone health and osteoporosis [23], Ca plays a role as a cofactor for blood coagulation and nerve and muscle function. Ca and Mg have opposing effects in that Mg acts as a Ca antagonist and can compete with Ca for protein and Ca transporter binding sites [17]. An imbalance of Ca and Mg in the blood may result in several clinical complications, including MetS, diabetes, hypertension, and CAD [24]. Low serum Mg levels or high serum Ca levels can pathologically contribute to CVD risk. Serum Ca/Mg ratios may be more characteristic of homoeostasis than measurements of serum Mg [25]. Furthermore, it is essential to maintain serum Ca and Mg levels within appropriate ranges, although these ranges for serum Ca/Mg ratios remain clinically uncertain [26]. For this reason and to better understand the Mg status in CF patients, we have hypothesized that abnormal serum Mg levels and inadequate Mg intake are frequent. Consequently, the main goal was to evaluate the serum and dietary Mg intake, the Ca/Mg ratios, and their association with nutritional parameters in these chronic patients.

## 2. Materials and Methods

### 2.1. Study Site, Design, and Participants

**Figure 1:**
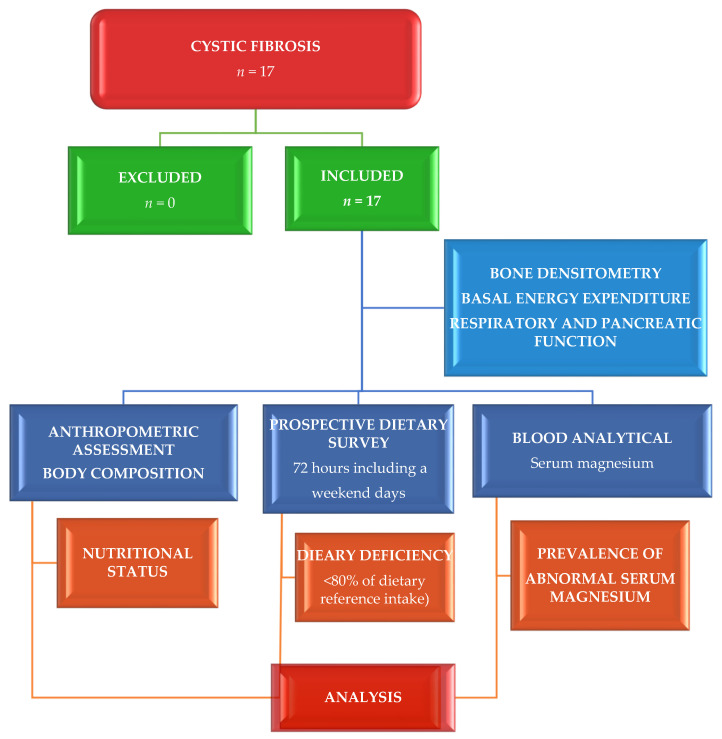
Flow diagram assignment of patients with cystic fibrosis (*n* = 17).

The design of this cross-sectional and observational study to evaluate Mg status, conducted in both pediatric and adult CF patients through its intake and serum levels, has been previously described [27,28]. It was carried out in the Nutrition Unit of the Pediatrics Service at the University Clinical Hospital in Valladolid, Spain, for 18 months. The inclusion criteria were patients with proven diagnosis of CF. Acute infection, hospitalization, and refusal to take part were exclusion criteria. The time of chronic diseases was shown in months (Figure 1). 

### 2.2. Ethical Consideration

The study protocol was reviewed and approved by the local ethics committee at the University Clinical Hospital (INSALUD-Valladolid, 14 February 2002), following the principles of the Declaration of Helsinki. The relatives/guardians of all the patients signed the informed consent before their participation.

### 2.3. Assessment of Phenotypical Characteristics

Data on age and gender were collected in a nutritional survey. Anthropometric assessment of weight, height, wrist, hip, waist, and mid-arm circumference was carried out using standard techniques. Z-score of weight-for-age, height-for-age, age-for-50° height, weight-for-height, BMI-for-age, BMI–height–age, and the mid-arm muscle area, fat-free mass, and fat mass were calculated using Frisancho [29] and Orbegozo tables [30]. Triceps, biceps, subscapular and suprailiac skinfolds were measured by standard methods with a Holtain Skinfold Caliper. Body composition was measured by anthropometry and bioelectrical impedance analysis (BIA) (RJL BIA-101 (RJL System, Detroit, MI, USA)). Ultrasound bone densitometry (DBM Sonic 1200 IGEA (Emsor S.A., Madrid, Spain)), bone mineral density (BMD), was measured by bone conduction velocity (BCS) of the last 4 fingers of the non-dominant hand [31]. Basal energy expenditure (EE) or resting EE (REE) was measured by fasting indirect calorimetry (IC) with a canopy system under standardized conditions (Deltatrac II (Datex-Ohmeda; Helsinki, Finland)).

### 2.4. Dietary Assessment

The participants measured and recorded all the food they ingested. Daily energy intake; fiber; carbohydrates; protein; lipids; monounsaturated, polyunsaturated, and saturated fats; vitamins A, B1, B2, B6, B12, C, D, E, niacin, and folic acid; and Ca, Mg, iron, zinc, and iodine were analyzed from the food consumption records of a 72 h prospective diet survey (including one of the weekend days) the week before the blood test. Adequate nutrient intake was assessed by the percentage of Dietary Reference Intake (%DRI) using the Mataix Food and Health software, which provided the percentage of actual nutrient intake with respect to Spanish recommendations [32,33]. Less than 80% DRI and more than 120% DRI were the cutoffs used to categorize insufficient or elevated dietary intake, respectively. In addition to pancreatic enzyme replacement therapy (PERT) and fat-soluble vitamin supplements, patients receive no additional Mg or Ca supplementation.

### 2.5. Clinical Evaluation

During the clinical examination of each patient, the presence of symptoms due to altered Mg status, such as early clinical symptoms of Mg deficiency (loss of appetite, nausea and vomiting, fatigue, and weakness), was assessed [34]. Furthermore, we evaluated whether the patients had any severe symptoms of hypomagnesemia: neuromuscular symptoms such as muscle weakness, tremors, seizures, and paresthesias; cardiovascular abnormalities such as arrhythmia, ventricular fibrillation, and hypertension; and metabolic abnormalities such as hypokalemia and hypocalcemia [10,35].

### 2.6. Laboratory Exploration

Fasting venous blood samples were collected to estimate serum Ca and Mg by standardized methods. The cutoff points for normal serum Mg level are 1.7 to 2.3 mg/dL (0.7 to 0.95 mmol/L). The following cut-off points were used for serum Ca in children (8.8–10.8 mg/dL) and adults (9–10.5 mg/dL) [36] and for Mg: symptomatic hypomagnesemia (<1.22 mg/dL), asymptomatic hypomagnesemia (1.22–1.82 mg/dL), CLMD (1.82–2.07 mg/dL), interval for health (2.07–2.32 mg/dL), asymptomatic hypermagnesemia (2.32–4.86 mg/dL), symptomatic hypermagnesemia (>4.86 mg/dL) [37]. If the serum albumin level was <4.0 g/dL, serum Ca was corrected using the following formula: Ca corrected (mg/dL) = measured Ca (mg/dL) + (4 − albumin (g/dL)) [38]. The cutoff for Ca/Mg intake ratio was 1.70–2.60 [39], and serum Ca/Mg ratio was 3.91–4.70 [26]. 

Complete blood count, complete biochemical, and acute-phase proteins: C-reactive protein (CRP) > 4 U/L and erythrocyte sedimentation rate (ESR) in women > 20 mm/h and men > 15 mm/h, were measured using standardized methods. Serum prealbumin ≤ 18 mg/dL, albumin ≤ 3.5 g/dL as visceral protein reserve, transferrin ≤ 200 mg/dL, lymphocytes < 2000 cell/mm^3^, total cholesterol (TC) > 200 (mild–moderate risk) and >225 mg/dL (high risk), and low-density-lipoprotein cholesterol (LDL-C) > 115 (mild–moderate risk) and >135 mg/dL (high risk) were used as cutoffs to evaluate abnormal values. Serum levels of folic acid; beta-carotene; vitamins B12, C, D, E, calcium, phosphorus, iron, zinc [40], and copper [41]; total immunoglobulin (Ig) G, IgG1–4, IgA, IgM, and IgE; C3 and C4 complement; CD3, CD4, CD8, CD16+56, CD19 lymphocytes, and CD4/CD8 ratio; and insulin-like growth factor-1 (IGF-1) and insulin-like growth factor-binding protein 3 (IGFBP3) were assessed.

### 2.7. Statistical Analysis

The main variables studied were serum Mg and dietary Mg intake. Anthropometric, biochemical (serum Ca and Ca/Mg ratios), dietary (Ca/Mg intake ratio), body composition, bone densitometry, and basal EE were secondary variables. The distribution of anthropometric results (quantitatively and Z-scores) and biochemical data were shown as mean, median, quartiles, SD, and range. Comorbidities are expressed as percentages. A comparison between groups for continuous and categorical variables was performed using the Mann–Whitney U and McNemar’s tests, respectively. Spearman’s correlation was performed to test the associations. The analysis of variance (Kruskal–Wallis test) was used to search for interactions. In order to determine if two qualitative variables are independent, we used the Fisher’s exact test (FET). To estimate the magnitude of the association between exposure and disease, we calculated odds ratios (OR). Simple and multiple linear regression analysis were calculated to study the relationships between two or more correlations. The analyses were performed using IBM SPSS version 24.0 (IBM Corp., Armonk, NY, USA). The significance level was established at *p* < 0.05 * and <0.01 **.

## 3. Results

Table 1 shows the baseline clinical characteristics of the participants (10 females, 59%) [27,28]. The mean age was 15 years (seven children, five adolescents, and five adults). The most frequent mutation was the homozygous Delta F580 (41%). A total of 29% of the subjects had undernutrition. There were two patients with overweight (8- and 13-year-old) and obesity (2- and 25-year-old) by waist-for-height index but not by BMI. The mean serum Ca (9.8 mg/dL or 2.45 mmol/L) and Mg (2.0 mg/dL or 0.82 mmol/L) were normal. A total of 47% had a serum Mg deficiency: two subjects had asymptomatic hypomagnesaemia with regular and deficient Mg intake, respectively, and six patients had CLMD, one with a low Mg intake, two with regular ingestion, and three with a high Mg intake. No patients had hypermagnesemia and abnormal serum Ca. 

The mean dietary intake of Ca (126% DRI: Dietary Reference Intake) and Mg (125% DRI) were high. A total of 41 and 47% of the patients had high Ca and Mg intakes, respectively, 12% had a dietary Mg deficiency, and 41% had adequate Mg consumption. Only one patient had CLMD and a poor Mg intake. A total of 24% had normal levels of Mg in the serum and diet. A total of 47 and 82% of the subjects had a high serum Ca/Mg ratio of >4.70 and a low Ca/Mg intake ratio of <1.70, respectively. A total of 87.5% of the patients with a high serum Ca/Mg ratio had at the same time a serum Mg deficiency. The likelihood of a high Ca/Mg ratio was 49 times (*p* = 0.010) higher among patients with a serum Mg deficiency than among those with normal serum Mg (Table 2). Therefore, 53% of our series had a high risk of Mg deficiency. 

No significant differences in the serum levels and dietary intake of Ca and Mg, serum, and dietary Ca/Mg ratios according to gender, abdominal ultrasound, and ERS were discovered (Table 3). Colonized patients had a lower Mg intake (*p* = 0.009) and higher Ca/Mg intake ratio (*p* = 0.008) than non-colonized ones. The mean basal EE was lower than the theoretical (*p* = 0.001) but was adequate along with the World Health Organization’s recommendation (*p* = 0.074). Eutrophic patients had a higher iron intake than undernutrition ones (*p* = 0.045). The nitrogen balance was higher in patients with a normal CRP than in patients with an abnormal CRP (*p* = 0.034). There were no differences in the anthropometric measures by gender and pancreatic and pulmonary function. Sufficient pancreatic (SP) patients had a lower BMI (*p* = 0.020) and a higher nitrogen balance than insufficient pancreatic (IP) patients. Lung function was not significantly worse in 18% of the patients colonized by *Pseudomonas aeruginosa*, *Candida* spp., and 23.5% by *Staphylococcus aureus*, compared to those without such colonization. There was a significant difference between the CF patients with IP (65%) and a regular abdominal ultrasound and those with SP (23.5%) and an abnormal abdominal ultrasound (*p* = 0.0001). Nitrogen intake (*p* = 0.000) and nitrogen balance (*p* = 0.034) were significantly higher in insufficient respiratory (IR) patients than sufficient respiratory (SP) patients. 

**Figure 2:**
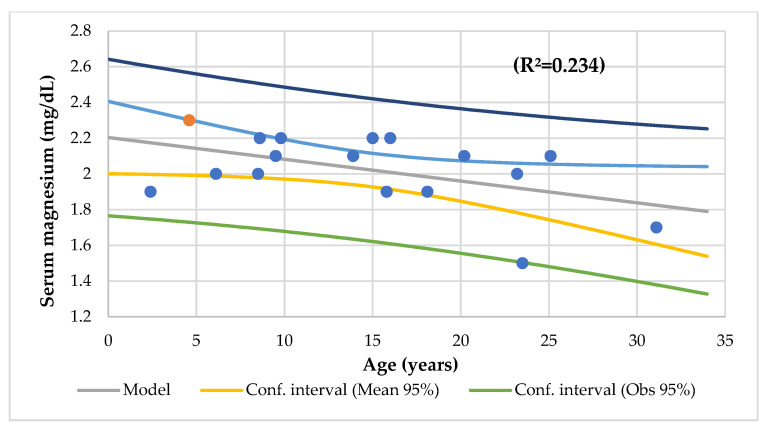
Regression serum magnesium (mg/dL) by age (years).

**Figure 3:**
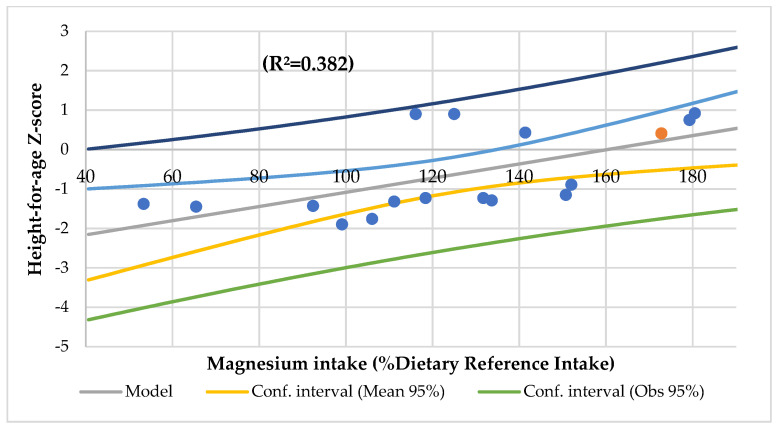
Regression height-for-age Z-score by magnesium intake (%Dietary Reference Intake).

Table 4 displays the linear and multilinear regression analyses. The serum Mg level had a significant association with age (Figure 2), serum phosphorus (Figure 3), serum Ca/Mg ratio, creatinine, alkaline phosphatase, basophils, and lymphocytes CD8 and a meaningful association with serum iron and alkaline phosphatase (R^2^ = 0.590), vitamin C, protein, iron, vitamin B12, polyunsaturated fat intake (R^2^ = 0.907), and serum Ca and Ca/Mg ratio (R^2^ = 0.986). 

**Figure 4:**
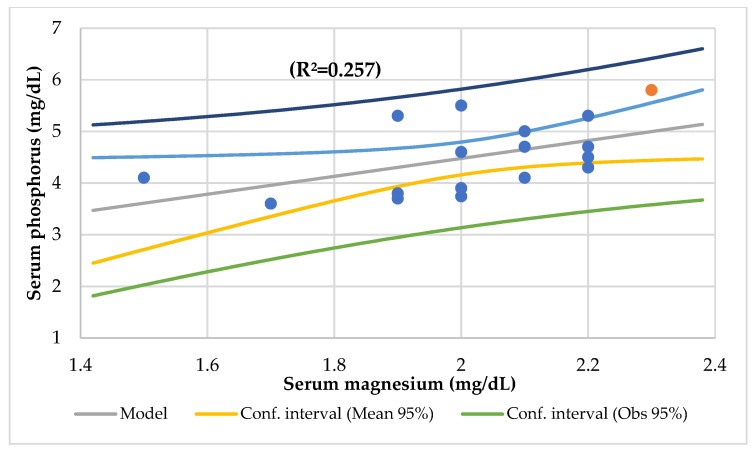
Regression serum phosphorus (mg/dL) by serum magnesium (mg/dL).

The serum Ca was associated with kilograms of fat mass by BIA, insulin-like growth factor-binding protein 3 (IGFBP3), transferrin, beta-carotene, total serum proteins (Figure 4), mean corpuscular hemoglobin (MCH), platelets, and urine nitrogen, and it had an association with urine nitrogen and EE (R^2^ = 0.656) and eosinophiles and the mean corpuscular volume (MCV) (R^2^ = 0.508). 

**Figure 5:**
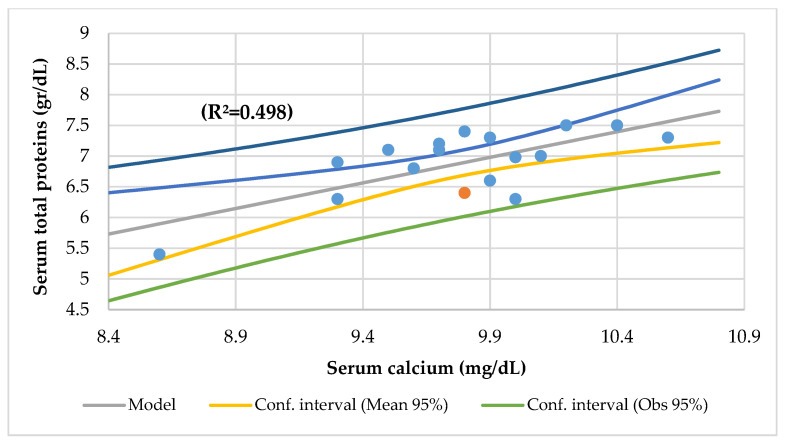
Regression serum total proteins (g/dL) by serum calcium (mg/dL).

**Figure 6:**
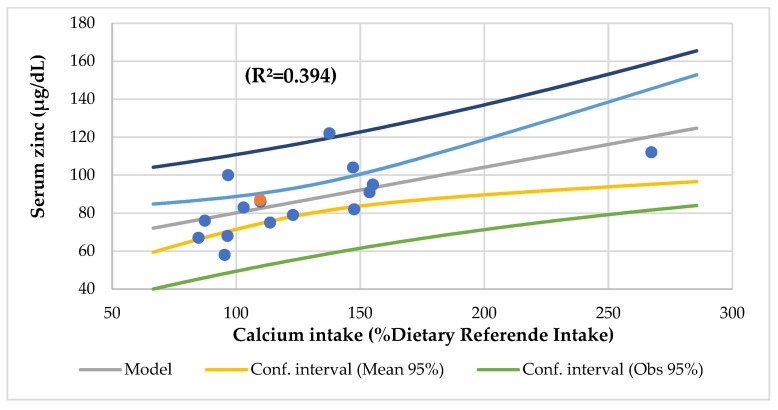
Regression serum zinc (µg/dL) by calcium intake (%Dietary Reference Intake).

The serum Ca/Mg ratio had an association with creatinine, serum vitamin B12, IgM (Figure 5), and urine creatinine and phosphorus, and it had an association with energy, iron, and vitamin C intake (R^2^ = 0.686), and serum Mg and Ca (R^2^ = 0.985). The Mg intake was associated with the height-for-age Z-score (Figure 6), Waterloo II, energy, protein, total lipids, folic acid, and zinc intakes, the Ca/Mg intake, transferrin saturation index, and serum vitamin E. 

**Figure 7:**
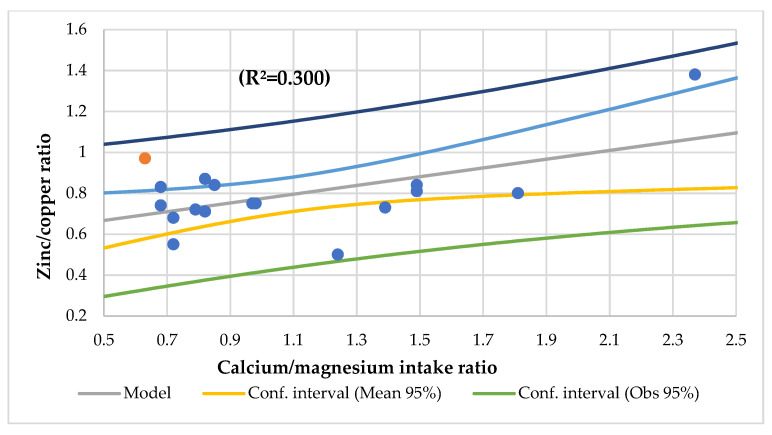
Regression zinc/copper ratio by calcium/magnesium intake ratio.

The Mg consumption had an association with the Waterloo and head circumference (R^2^ = 0.712), total lipids, vitamin B12 and calcium intake (R^2^ = 0.906), and total lipids and zinc intake (R^2^ = 0.682). The Ca intake had a significant association with the suprailiac skinfold Z-score, nutritional index, serum zinc (Figure 7), and transferrin. The Ca had an association with the platelet and eosinophiles (R^2^ = 0.615) and with serum zinc and iron (R^2^ = 0.639). 

**Figure 8:**
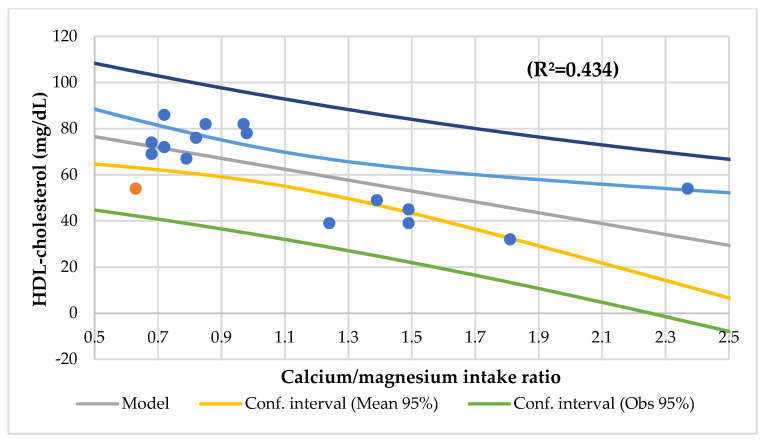
Regression HDL-cholesterol (mg/dL) by calcium/magnesium intake ratio.

The Ca/Mg intake ratio had an association with vitamin C, the vitamin B12 niacin intake, the zinc/copper ratio, the transferrin saturation index, and HDL-cholesterol (Figure 8) and an association with the niacin and folic acid intake (R^2^ = 0.628) and vitamin B12 and niacin intake (R^2^ = 0.627). 

## 4. Discussion

Interestingly, not much is known about the Mg status in CF patients. To the best of our knowledge, this is the first study to explore the Mg status through serum levels and the dietary intake of Ca and Mg and the Ca/Mg ratios concerning nutritional indicators in CF patients. In this series, the results showed that the mean serum Mg and Ca were standard and the dietary Mg and Ca intakes were high. A total of 47% had a serum Mg deficiency. No patients had hypermagnesemia and an abnormal serum Ca. A total of 41 and 47% of the patients had a high dietary Ca and Mg consumption, respectively, 12% had a dietary Mg deficiency, and 41% had an adequate Mg consumption. Only one patient had CLMD and an inadequate Mg intake. A total of 24% had normal levels of Mg in the serum and diet. A total of 47 and 82% of the participants had a high serum Ca/Mg ratio of >4.70 and a low Ca/Mg intake ratio of <1.70, respectively. A total of 87.5% of the patients with a high serum Ca/Mg ratio had, at the same time, a serum Mg deficiency. The likelihood of a high Ca/Mg ratio was 49 times higher among patients with a serum Mg deficiency than among those with a normal serum Mg. Colonized patients had a lower Mg intake and a higher Ca/Mg intake ratio than non-colonized ones. The regression analyses display the meaningful association between the serum Ca, Mg, diet Ca and Mg intake, and Ca/Mg ratios with several nutritional indicators. Therefore, 53% of our series had a high risk of a Mg deficiency.

### 4.1. Deficient Magnesium Status

It is relevant to consider that, although a high rate of Mg deficiency is currently postulated [37,42] for up to 20% of the general population [43], reference ranges suggested that it could significantly improve public health [39] by identifying the population at risk of a marginal or subclinical Mg deficiency [37,43]. In children, Pagana recommended limits of 0.69 to 0.85 mmol/L (1.70 to 2.06 mg/dL) for the serum Mg [36]. In adults, a reference range of 0.75 to 0.95 mmol/L (1.82 to 2.31 mg/dL) can often be found [37]. Serum Mg values of <0.7 mmol/L (1.7 mg/dL) indicate a Mg deficiency [44]. Critical reference values are <0.5 mmol/L (1.0 mg/L) and >2.0 mmol/L (4.9 mg/dL) [45]. According to the current epidemiological data, the risk of several diseases increases with a decreasing serum Mg, even within the reference range of 0.75 to 0.95 mmol/L [39,46]. A subclinical magnesium deficiency may exist despite presumed normality within the previous reference interval [37]. The German Society for Magnesium Research e.V. proposed a precautionary lower limit value of 0.80 mmol/L (1.94 mg/dL) [47]. At the international level, according to several authors, the target value should be a serum Mg of <0.85 mmol/L (2.07 mg/dL) [39,46,48,49]. 

This study found that a mean serum Mg of 2.0 mg/dL (0.8 mmol/L) was normal and that the levels decreased significantly with age (Figure 2). As in our series, Santi reports that the serum Mg levels decreased with age and found hypomagnesemia in more than half of the patients with advanced CF [50]. In addition, using the reference ranges suggested by Costello [37], 47% of the participants with a mean age of 14.8 years old (range 2 to 31 years) had a serum Mg deficiency. Two subjects (12%) had asymptomatic hypomagnesemia (1.82 mg/dL or <0.75 mmol/L) and six patients (35%) had CLMD (2.07 mg/dL or <0.85 mmol/L). Similarly, Gupta et al. (2007) reported that 57% of the 106 CF transplanted patients (mean age 24.5 years, mean Mg level of 1.8 mg/dL) had hypomagnesemia. This condition may be a risk factor for post-transplant complications, including convulsions [10]. According to Lin et al., patients with a moderate serum Mg within the reference range (0.82–0.95 mmol/L) had the lowest mortality risk, and patients with low serum Mg levels (0.39–0.81 mmol/L) had the highest mortality risk. High serum Mg levels (0.96–2.28 mmol/L) were associated with higher CAD mortality [26]. Therefore, our patients may suffer an increased risk of morbidity and mortality due to the current data suggesting an augmented risk of CVD, T2D, other chronic diseases, and mortality from these diseases, even at values below 0.75 to 0.85 mmol/L [37].

It is interesting to find that there were no patients with hypermagnesemia in our series. Unlike hypomagnesemia, hypermagnesemia is rare [51]. This circumstance is caused by a severe renal insufficiency or excessive Mg intake [52]. In CF, the true prevalence of hypomagnesemia is unknown [10]. This deficiency is mainly due to four factors: (1) insufficient dietary intake, (2) reduced intestinal absorption due to disease-associated pancreatic insufficiency, (3) CF-associated hyperglycemia (CF-related diabetes, CFRD) [53] that can trigger hypomagnesemia and emaciation-associated kidney disease [10], and (4) medications such as B2 agonists, aminoglycoside antibiotics, amphotericin B and tobramycin [50], diuretics (furosemide, thiazide) [54], epidermal growth factor receptor inhibitors (cetuximab), calcineurin inhibitors (tacrolimus [55] and cyclosporine A [50]), cisplatin, and some antimicrobials (rapamycin, pentamidine, foscarnet) [56]. Hypermagnesemia remains asymptomatic up to a serum Mg of 2.00 mmol/L (4.86 mg/dL) [17,57]. Contrariwise, the symptoms of a Mg deficiency are often not experienced until levels are <0.5 mmol/L (1.21 mg/dL) [58]. It is not uncommon for patients to present with no signs or symptoms of this deficiency, even with severe hypomagnesemia and if the serum concentration gradually declines [59]. In our series, CF patients did not present any symptoms related to a deficient Mg status.

Results show that the average diet of our series was high in protein with an adequate intake of nutrients, except for the low iodine intake [27,28]. One of the most relevant causes of hypomagnesemia, like other elements, is insufficient dietary intake. In fact, several studies show that most of the European and North America population consumes less than the recommended daily allowance (RDA) of Mg [60,61]. Nevertheless, in our series, although the mean dietary intake of Mg was high, and only 12% had a Mg intake below the proposed cut-off point, 47% of the participants had a serum Mg deficiency (Table 2). Out of 47% of the patients who had an elevated Mg intake (eight cases), four had a serum Mg deficiency (23.5%). In addition, out of 41% of the patients who presented an adequate Mg diet (seven cases), three had a serum Mg deficiency (17.5%), and only one patient (6%) had an inadequate consumption of Mg and CLMD. The other patient with a low Mg intake had a normal serum Mg (6%). The chronic low dietary intake of Mg leads to a serum and intracellular Mg deficiency, especially evident in obese people with MetS, elders, and non-white people with insulin resistance [62]. Studies have also demonstrated that a reduced risk of several chronic diseases is observed with a higher Mg intake or supplementation [37].

Importantly, a subclinical Mg deficiency does not manifest as clinically apparent symptoms. Therefore, it is not easily recognized by the clinician [19]. In CF patients, it is important to measure it and regularly control the Mg status because of the longevity increase and the burden and prevalence of the comorbidities increase, which includes CFRD, CF-related liver disease (CFLD), and CF-related kidney (CFKD) and bone disease (CFBD), along with the increased chance for obesity and overweight, which were all reported in CF patients [9,63,64]. Over-nutrition in CF patients, especially those with pancreatic insufficiency, is a relatively new, emerging phenomenon. Hanna et al. reported that 23% of their patients with CF were overweight or obese [65]. However, macrovascular disease, such as CAD, is very rare in patients with CF, probably due to the previously low median survival age in this patient population. It is possible that more cardiovascular and macrovascular complications will be observed over time [66]. Furthermore, CF patients receiving courses of aminoglycosides should have regular monitoring of their serum Mg levels. There should also be awareness that people with CFRD or those on prolonged courses of PPI may be at an increased risk of a Mg deficiency [67,68]. 

### 4.2. Calcium/Magnesium Ratios

The serum Mg is one main factor in the delayed progression of atherosclerosis [69] with a negative effect on endothelial function in patients with a Mg deficiency. In normal adults, a lower level of Mg is related to CAD [70]. An elevated serum Ca level is an independent risk factor for mortality in patients on their initial hemodialysis (HD) [71]. An increased concentration of ionized Ca launches and promotes coagulation cascades, leading to vessel calcification, causing CVD, peripheral arterial disease, and ischemic and hemorrhagic strokes [69]. In this study, the mean serum Ca (9.8 ± 0.5 mg/dL) was normal, and the mean dietary Ca intake (126%DRI) was high. A total of 41% of the subjects had a high Ca intake. No patients had an abnormal serum Ca or poor Ca intake. In a study performed on 24 CF adults during the pulmonary exacerbation, the subjects exhibited a higher prevalence of Ca deficiency [72]. CF patients experience the overactivation of inflammatory processes, including increased Ca signaling [73]. An abnormal Ca profile in CF cells (including airway epithelial cells and immune cells) can impact cell function, viability, and susceptibility to pathogens [74]. This abnormal profile observed in CF is initially caused by intrinsic defects associated with the CFTR mutation [75]. Calcium intake and excretion in the human body are tightly regulated to maintain a constant extracellular and intracellular concentration [76].

Surprisingly, there are no functional biomarkers for Mg status, such as ferritin for iron status [77]. Because the serum Mg has a limited informative value [48] due to a poor correlation between the serum and intracellular [78] or whole-body Mg concentrations [59], and they measure short-term variations in the intake [77], other techniques or biomarkers such as a Ca/Mg ratio are essential. An increased cellular Ca/Mg ratio is a potentially pathogenic factor in the development of arteriosclerosis and hypertension because a Mg deficiency increases the progress of atherosclerosis [79]. Based on a study of healthy Chinese women of childbearing age (18–44 years), a reference range of 2.41 to 3.44 was suggested [80]. A case–control study informed an increased Mg and a decreased Ca/Mg ratio in the whole blood correlated with MetS [81]. Alternatively, a moderate Ca/Mg ratio (3.91–4.70) had the lowest mortality risk [26]. In addition, a high Ca/Mg ratio of 4.37 was significantly associated with all-cause and CVD mortality in incident dialysis patients [25]. In our study, the mean serum Ca/Mg ratio was 4.89 ± 0.54 (4.26 to 6.20). Based on the cutoff of >4.70, 47% of the patients (8 cases) had a high serum Ca/Mg ratio, and simultaneously, 87.5% of these patients had a low serum Mg level (FET, *p* = 0.005). The likelihood of a high Ca/Mg ratio was 49 times higher (*p* = 0.010) in patients with a serum Mg deficiency than in the normal serum Mg patients. Thus, these results support the high risk in our series of developing chronic diseases such as CVD. 

A daily intake (DI) of 3.6 mg/kg is necessary to maintain a Mg balance in humans [82]. Low Mg intakes along with high Ca intakes and elevated Ca/Mg intake ratios were associated with an increased risk of multiple chronic conditions [83] such as CAD [84], CVD and Mets [83], and acute myocardial infarction (AMI) [84] (biomarker) [85], in addition to some cancers (colorectal [86], prostate [87], esophageal [88]), and total mortality, as well as vitamin D status [89]. Data from the National Health and Nutrition Examination Survey (NHANES) showed that the mean Ca/Mg intake ratio from foods alone for US adults was >3.00 since 2000 [39]. A high (>2.60) or low (<1.70) dietary Ca/Mg intake ratio may modify the associations between their intakes and the risk of various outcomes, including gastrointestinal neoplasia [86,90,91] and cancer mortality [92]. Suggesting a Ca/Mg ratio range between 1.7 and 2.6 (weight to weight) [39] is optimal for the Ca intake for these results [86,90,91,92]. Current evidence suggests that a reduction in disease risk can occur with a dietary Ca/Mg ratio between 1.70 and 2.60 and that these benefits may rely on gender and the specific health outcome [39]. A ratio of >1.70 would be associated with the reduction in total mortality in men [92] as well as a lower risk of advanced adenoma and distal colorectal cancer [93].

A high dietary Ca/Mg ratio (>2.60) may affect the body Mg status, whereas a high Mg intake could negatively affect individuals with an exceedingly low dietary Ca/Mg ratio (<1.70) [39]. Increased Mg was associated with a reduced risk of noncardiac gastric carcinoma [88]. However, a high Mg intake was associated with an increased risk of gastric cancer mortality, particularly in patients with a Ca/Mg ratio of ≤1.7 [92]. In our series, the mean Ca/Mg intake ratio was 1.10 ± 0.49 (0.63 to 2.37), and 82% of the patients had a Ca/Mg ratio of <1.70. Only two patients were within the reference ranges of 1.70 to 2–60 [92]. A 23-year-old eutrophic man with CLMD and normal serum Ca levels had a normal dietary Ca intake and an insufficient Mg consumption. Another 15-year-old undernourished adolescent male with normal serum Ca and Mg levels had a high Ca intake and inadequate Mg consumption. People with physical activity and a dietary Ca/Mg ratio between 1.70 and 2.60 showed a reduced risk of death from cancer [94] and improved cognitive function [95]. This high percentage of our series with a Ca/Mg intake ratio of <1.70 may have a higher risk of esophageal adenocarcinoma [88] and an increased risk of total mortality in women [92]. However, simultaneously, this high percentage of patients with a low Ca/Mg ratio could have a decreased risk of colorectal adenoma (≤2.78) [90] and colorectal adenoma recurrence [86]. In addition, a higher intake of Mg-rich foods (whole grains, nuts and seeds, legumes, and dark green vegetables) is related to a lower incidence of obesity, T2D, and MetS [96], which may be the key to the high dietary Mg intake in our series. 

Interestingly, in this study, the dietary Mg intake was significantly higher in non-colonized patients than in colonized ones (*p* = 0.009), especially in the group of patients of ≥10 years (*p* = 0.034). This difference may be due to a lower energy and micronutrient intake in colonized patients. In contrast, in 102 children with CF (ages 2 to 18 years), an increased intake of most micronutrients was caused by a higher EI, with high school children at the greatest risk of inadequate Mg consumption [97]. A Mg deficiency related to inflammation and oxidative stress [98] may exacerbate the course of COVID-19, suggesting a permanent control of the state of Mg and, where appropriate, its supplementation [99]. Leading evidence supports the use of Mg in the prevention and treatment of many common health conditions such as migraine headache, MetS, diabetes, hyperlipidemia, asthma, premenstrual syndrome, preeclampsia, and various cardiac arrhythmias [100]. Mg supplementation, which is safe and cost-effective, helped [55] and improved both the Shwachman–Kulczycki (SK) score and respiratory muscle strength in pediatric patients with CF [101]. However, a high dose of Mg can cause gastrointestinal side effects, especially diarrhea, particularly in patients taking higher dose Mg supplements after lung transplantation [55].

### 4.3. Nutritional Status

Regarding nutritional status, BMI may also affect Mg status, particularly in women and children [102]. In this series of CF patients, between children and adults, 29% had undernutrition. Surprisingly, based on the nutritional status by BMI, there was no significant difference in the prevalence of cases with a dietary and serum Mg deficiency and in the Ca/Mg ratios. Furthermore, the PS patients had a lower BMI and a higher nitrogen balance than the PI ones (*p* = 0.020). However, the nitrogen intake and balance were significantly higher in the IR patients than in the SR ones. In addition, the patients with an elevated CRP had a lower nitrogen balance than the patients with a normal CRP. Although the prevalence of malnutrition in the patients with CF decreases significantly, figures around 25% happened in both adults and children [5], as per our study. We must consider that malnutrition is a predictive risk factor for morbidity and mortality in this disease. A parallel decrease in the nutritional status and respiratory function would negatively affect the prognosis and quality of life [103]. Kilinc et al. reported that well-nourished patients had significantly higher lung function test scores than the other undernourished group [104]. Although in our study we did not have obese patients by BMI, there were two overweight patients (8 and 13 years) and obesity (2 and 25 years) by the waist–height index. Obese patients have a lower Mg intake and a reduced Mg status than non-obese age-matched controls [102]. 

Magnesium deficiency should be considered a nutrient of significant concern for health and well-being [105]. Even though we know that, during child growth, there is a retention of Mg of 3 mg/kg of body weight per day [106], few studies discuss the relationship between Mg intake and child growth. Protein and micronutrients such as zinc, potassium, Mg, and vitamin D affect growth hormones and IGF-1, which are involved in the critical points of osseous growth and development [107]. Likewise, Ca and phosphorus play an essential role in the growth and development of bone mass, which is at a maximum in the pediatric stage [108]. Although the serum Ca was associated with fat mass by BIA (R^2^ = 0.789) and IGFBP3 (R^2^ = 0.332), there was no association between the serum Mg and Ca/Mg ratios and anthropometric parameters. In addition, the dietary Mg intake was associated with a height-for-age Z-score (R^2^ = 0.444) (Figure 3) and Waterloo II (R^2^ = 0.465) and had a meaningful association with Waterloo and head circumference (R^2^ = 0.418). The Ca intake was associated with a suprailiac skinfold Z-score (R^2^ = 0.418) and nutritional index (R^2^ = 0.262). In a follow-up study of 265 healthy newborns for two years, dietary Mg intake was associated with the length-for-age Z-score at 12 months [107]. 

### 4.4. Magnesium and Its Associations

Remarkably, few studies link Mg with other micronutrients in CF patients. Nevertheless, in this study, we found several interesting and significant associations that we should consider. For example, the serum Mg had a significant association with vitamin C, protein, iron, vitamin B12, and polyunsaturated fat intake (R^2^ = 0.907). The serum Ca had an association with beta-carotene (R^2^ = 0.557) and the serum Ca/Mg ratio with energy, iron, and vitamin C intake (R^2^ = 0.686) and serum vitamin B12 (R^2^ = 0.388). The magnesium intake had a meaningful association with energy, protein, total lipids, folic acid, zinc and Ca/Mg intake ratio, and serum vitamin E (R^2^ = 0.359) and a significant association with total lipids, vitamin B12, Ca intake (R^2^ = 0.906), and total lipids and zinc intake (R^2^ = 0.682). Although the serum Ca and dietary Ca consumption did not have associations with any nutrients, the Ca/Mg intake ratio had a meaningful association with the vitamin C, niacin, and vitamin B12 intake, with niacin and folic acid (R^2^ = 0.628), and with the vitamin B12 and niacin intake (R^2^ = 0.627).

Vitamins A, E, and C are known to be powerful non-enzymatic antioxidants [109]. Vitamin C (l-ascorbic acid) in high concentrations can kill cancer cells through a pro-oxidant property [110]. Moreover, Mg supplementation increased the cellular absorption of vitamin C, improving its anticancer effects in both in vitro and in vivo systems by the sodium-dependent vitamin C transporter-2 family (SVCT-2) activation [111]. Alternatively, vitamin B12 is essential for maintaining a healthy gut microbiome. Furthermore, the combination of vitamin B12, D, and Mg in elders with COVID-19 reduced the proportion of patients with clinical deterioration who required oxygen support, intensive care support, or both [112]. Iron is a pro-oxidant nutrient enhancing the production of reactive oxygen species and the synthesis of inflammatory markers [113]. In our series, the iron intake was significantly higher in eutrophic patients than in undernourished ones. In a case–control study (27–59 years), in the group of diabetes patients, an inverse correlation between erythrocyte Mg and serum iron was observed [114]. In T2D patients, some studies suggested that a Mg deficiency and iron metabolism are involved in the induction of hemolysis, which contributes to iron release [113]. Folic acid is an essential micronutrient for many physiological processes [115]. In an in *vitro study* in rats, added extracellular Mg and folic acid decreased the homocysteine-induced matrix metalloproteinase-2 (MMP-2), which plays an essential role in the formation and progression of atherosclerotic lesions secretion [116]. 

In this study, the serum Mg was associated with the serum phosphorus (R^2^ = 0.273) (Figure 4), serum Ca/Mg ratio (R^2^ = 0.807), creatinine (R^2^ = 0.477), and alkaline phosphatase (R^2^ = 0.293) and an association with the serum iron and alkaline phosphatase (R^2^ = 0.590) and the serum Ca/Mg ratio and Ca (R^2^ = 0.986). The serum Ca had a meaningful association with the serum total proteins (R^2^ = 0.498) (Figure 5). The serum Ca/Mg ratio had an association with creatinine (R^2^ = 0.686) and serum Mg and Ca (R^2^ = 0.985). The calcium intake had an association with serum zinc (R^2^ = 0.394) (Figure 6) and serum zinc and iron (R^2^ = 0.639). The Ca/Mg intake ratio had an association with the zinc/copper ratio (R^2^ = 0.327) (Figure 7) and HDL-cholesterol (R^2^ = 0.434) (Figure 8). Alkaline phosphatase is a marker of bone turnover [117]. In 120 patients with dialysis CKD, hypomagnesemia increased the alkaline phosphatase and parathormone levels [118]. In an animal study, the Mg loading had no consistent effect on the serum alkaline phosphatase [117]. In a series of chronically ill children, the malnourished patients had a significant association between the dietary intakes of zinc and Mg [119]. Nielsen pointed out the possible interaction between Mg and zinc because a marginal zinc deficiency decreased the Mg excretion and increased the Mg concentration in bone [40,120]. Furthermore, the co-supplementation of Mg and zinc was beneficial for patients with DM2 and CHD, improving fasting plasma glucose (FPG), HDL-cholesterol, CRP, and insulin [121]. The zinc/copper ratio was significantly associated with renal function in all subjects and glycemic control in patients with T2D [122].

We must consider that cystic CFRD is a distinct form of diabetes associated with significantly higher morbidity and mortality in the CF population [123]. It is important to control a Mg deficiency as it is related to the development of lipid abnormalities and related disorders, such as MetS, T2D, or CVD [124]. Additionally, a higher Mg intake produces beneficial increases in the HDL concentrations [125]. In 210 patients with T2D, a positive relationship was found between the Mg intake and HDL-cholesterol (*p* = 0.005) and with energy intake (*r* 0.520; *p* = 0.000) [126]. In 57 patients with grade 3 diabetic foot ulcer (DFU), co-supplementation with Mg and vitamin E for 12 weeks had beneficial effects on ulcer size, glycemic control [127], triglycerides, LDL- and HDL-cholesterol, high sensitivity C-reactive protein (hs-CRP), total antioxidant capacity (TAC), and malondialdehyde (MDA) levels [128]. This combination effectively removes the peroxyl radical from cell membranes by inhibiting lipid peroxidation [127]. In a randomized, single-blind, controlled study (400 patients aged 25 to 63 years), 206 subjects took a Mg-rich diet. Therefore, the dietary Mg intake may have contributed to a reduction in the serum total cholesterol, LDL cholesterol, and triglycerides and a marginal increase in the HDL-cholesterol [129]. 

The finding showed that transferrin had a meaningful association with the serum Ca (R^2^ = 0.240) and dietary Ca intake (R^2^ = 0.345), and the transferrin saturation index had an association with the dietary Mg intake (R^2^ = 0.397) and Ca/Mg intake ratio (R^2^ = 0.250). Transferrin is a blood-plasma glycoprotein that plays a central role in iron metabolism and is responsible for ferric-ion delivery [130]. In an animal study, the concentration of the transferrin receptor 2 (TfR2) showed an increasing trend in the liver of rats treated with Mg sulfate [131]. In addition, the Ca signaling through the CAMKK2 and CAMK4 (calcium/calmodulin-dependent protein kinase kinase) proteins affects transferrin protein-mediated iron transport [132]. Interestingly, the serum Ca had a significant association with the urine nitrogen (R^2^ = 0.387), and the serum Ca/Mg ratio had an association with the urine creatine (R^2^ = 0.270) and phosphorus (R^2^ = 0.298). We must know that urinary excretions of Mg, Ca, and phosphorus excretions vary significantly between populations due to dietary habits, physical activity, the mineral content of water, climate, genetics, and race [133]. However, in a case–control study, CF patients showed a higher urinary excretion of phosphate and oxalate and a lower citrate and magnesium elimination, which predisposes these patients to future urolithiasis [134].

**Figure 9:**
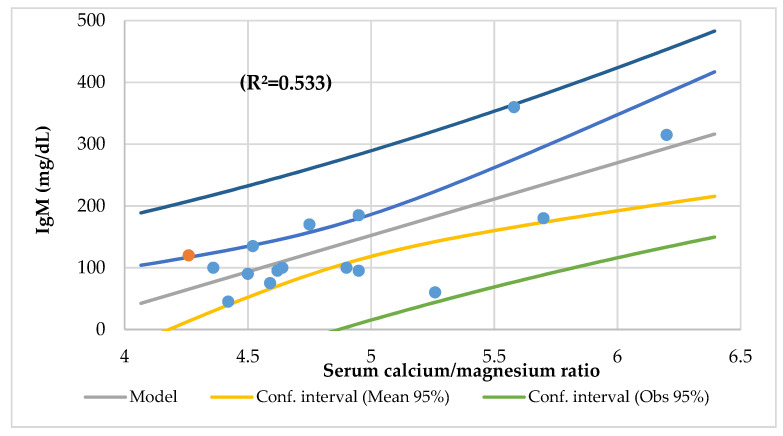
Regression IgM (mg/dL) by serum calcium/magnesium ratio.

Surprisingly, only one patient with a dietary Mg deficiency developed CLMD. Subclinical Mg deficiency is a common and underrecognized problem throughout the world [43]. Around 10–30% of a given population have a subclinical Mg deficiency based on serum Mg levels of <0.80 mmol/L [37]. It is caused by a low dietary intake often occurring in the population, and it is a predisposing factor for chronic inflammatory stress that is conducive for chronic disease [105]. In this study, the serum Mg was associated with basophiles (R^2^ = 0.365) and lymphocytes CD8 (R^2^ = 0.364). The serum Ca was associated with MCH (R^2^ = 0.281), platelets (R^2^ = 0.268), and eosinophiles and MCV (R^2^ = 0.508). The serum Ca was associated with IgM (R^2^ = 0.533) (Figure 9). The dietary Ca intake was associated with platelets and eosinophiles (R^2^ = 0.615). 

Magnesium is closely related to the immune system in both nonspecific and specific immune responses (i.e., innate and acquired immune responses) [135]. It has effects on the acute-phase response and the function of macrophages, for example, in their response to cytokines [136]. Mg has a major influence on the development, differentiation, and proliferation of lymphocytes [137]. It is important to underline that a moderate or subclinical Mg deficiency can induce a chronic low-grade inflammation sustained by the release of inflammatory cytokines and the production of free radicals, which exacerbate a preexisting inflammatory status [138]. For this reason, Mg depletion is considered a risk factor for pathological conditions characterized by chronic inflammation, such as hypertension and cardiovascular disorders but also MetS and diabetes [139]. In metabolic diseases, a low Mg status mainly due to unhealthy diets contributes to generate a pro-inflammatory environment that exacerbates metabolic derangement [140]. 

The proposed action of Mg in CF patients is not completely understood. Altered Mg levels can affect DNA/RNA stabilization, modulation of enzymatic activities, regulation of ion channel function, and protection of the cell against oxidative stress and contribute to pathological conditions [17]. The ATP binds to a Mg ion to compose a biologically functional form, where the majority of intracellular ATP and Mg form Mg-ATP complexes [141]. The ATP regulates the opening and closing of the CFTR Cl channel, and the Mg plays a role in many crucial enzyme systems, especially those involving ATP metabolism [142]. The energy of the CFTR-ATP-Mg interaction in the transition state is responsible for the opening of the CFTR ion channel rather than the energy of the ATP hydrolysis [143]. Furthermore, there is a functional link between CFTR channels and transient receptor potential (TRP) proteins in human airway epithelial cells, giving an increase in Ca entry and a decrease in Mg entry, both mediated by the TRP channels in mutated CFTR epithelial cells. This situation triggers the activation of Ca-gated chloride channels (CaCC) or CFTR itself through the activation of adenylyl cyclase, PKA, and tyrosine kinase. They also lead to an increased inflammatory response [144]. In this way, Mg may reduce the airway inflammation that underlies several lung diseases, including chronic obstructive pulmonary disorder (COPD) and cystic fibrosis [17]. Mg protects against inflammation by reducing oxidative stress, inhibiting substance P release, and preventing Ca toxicity by inhibiting voltage-gated Ca channels [145]. Moreover, Mg acts as a cofactor in the enzymatic degradation of DNA by rhDNase I, so a minimum concentration of Mg is required to obtain optimal rhDNase I activity [146]. Patients supplemented with Mg increased their maximal inspiratory pressure (MIP) and maximal expiratory pressure (MEP) [101]. Magnesium’s functions as a natural Ca antagonist, glutamate NMDA receptor blocker, vasodilator, and antioxidant and anti-inflammatory agent are responsible for its therapeutic benefits [147].

At this point, we need to consider several highlights. First of all, the mean serum Mg (2 mg/dL) and Ca (9.8 mg/dL) were normal. A total of 47% of the patients had a serum Mg deficiency (two patients had asymptomatic hypomagnesemia and six had CLMD). Secondly, the mean dietary Mg (125% DRI) and Ca (126% DRI) intake were high. A total of 41 and 47% of the patients had a higher Ca and Mg intake, respectively, and 12% had a dietary Mg deficiency. Only one patient with a dietary Mg deficiency had CLMD. Thirdly, 47 and 82% of the subjects had a high serum Ca/Mg ratio of >4.70 and a low Ca/Mg intake ratio of <1.70, respectively. The likelihood of a high Ca/Mg ratio was 49 times higher among patients with a serum Mg deficiency than among those with a normal serum Mg. Last but not least, this study demonstrated that serum Mg and Ca levels, dietary Mg and Ca intake, serum Ca/Mg ratio, and Ca/Mg intake ratio had a meaningful association with several of the nutritional parameters studied. Considering all the highlights, we should indicate that, although the dietary Mg intake was high, there were 53% of our series with a high risk of Mg deficiency and developing cardiovascular disease (CVD), type 2 diabetes (T2D), and metabolic syndrome (MetS), and even several cancers.

The results respond to the main aim of this study and indicate the need to continue studying the relationship between the nutritional status of patients with CF and an abnormal micronutrient status to understand the essential balance between both. A limitation of this study is the small number of participants. However, its strengths lie in assessing the serum and dietary Mg levels and their relationship with anthropometric, biochemical, and diet indicators. Despite the small number of patients, the in-depth analysis is justified by the lack of scientific literature on the theme. We suggest the implementation of multicenter trials to improve the knowledge of the Mg status in these patients and to determine the necessary and appropriate amount of Mg supplementation to improve the nutritional level in cystic fibrosis patients when necessary.

## 5. Conclusions

In this series of CF patients, the mean serum magnesium was normal, and the dietary magnesium intake was high. Only one patient had a chronic latent magnesium deficiency and inadequate magnesium intake. Two patients had a dietary Mg deficiency and 47% had a high daily Mg consumption. A total of 47% of the subjects had a high serum Ca/Mg ratio and 82% of the patients had a low Ca/Mg intake ratio. The likelihood of a high Ca/Mg ratio was 49 times higher among patients with a serum Mg deficiency than among those with a normal serum Mg. The serum magnesium concentration and dietary magnesium intake were associated with several nutritional biomarkers. Therefore, there were 53% of the CF patients at risk of a deficient Mg status and developing a chronic disease and even cancer. 

## Figures and Tables

**Table 1:** Baseline demographic and clinical characteristics of participants (*n* = 17 *) [27,28].

Characteristics	Mean ± SD or No. (%)	Median	Range
Age (years)	14.8 ± 8	15	2–31
Body mass index Z-score	−0.95 ± 1.1	−0.6	−3.8–0.6
Basal energy expenditure	1078 ± 303	1149	440–1490
Theoretical basal energy expenditure	2193 ± 576	2200	1066–3251
WHO basal energy expenditure	1185 ± 233	1230	598–1559
Serum magnesium (mg/dL)	2.0 ± 0.2	2.0	1.5–2.3
Serum calcium (mg/dL)	9.8 ± 0.5	9.8	8.6–10.6
Serum calcium/magnesium ratio	4.89 ± 0.54	4.69	4.26–6.20
Energy (calories)	2595 ± 464	2672	1846–3410
Magnesium intake (%DRI)	125 ± 37	125	53–180
Calcium intake (%DRI)	127 ± 44	112	85–267
Calcium/magnesium intake ratio	1.09 ± 0.49	0.9	0.63–2.37

* Seventeen cystic fibrosis patients were screened, included, and analyzed.

**Table 2:** Serum levels, dietary intake, and proportions of calcium and magnesium in patients with cystic fibrosis according to nutritional status (*n* = 17 *).

Gender by Nutritional Status (BMI)	Age (Years)	Serum (mg/dL)			Dietary Intake (%DRI)		
		Ca ^a^	Mg ^b^	Ca/Mg Ratio	Ca	Mg	Ca/Mg Ratio
Eutrophic							
Male	2	10.0	1.9 ^†^^†^	5.26 ^‡^	147 **	106	1.39 ^#^
Male	4	9.8	2.3	4.30	110	173 *	0.63 ^#^
Female	6	9.8	2.0 ^†^^†^	4.95 ^‡^	122 **	99	1.24 ^#^
Male	8	9.9	2.0 ^††^	4.51 ^‡^	267 **	179 **	1.49 ^#^
Male	8	9.6	2.2	4.36	154 **	180 **	0.85 ^#^
Female	9	10.4	2.1	4.95 ^‡^	138 **	92	1.49 ^#^
Female	9	10.1	2.2	4.59	84	118	0.72 ^#^
Male	13	9.7	2.1	4.62	109	134 **	0.82 ^#^
Female	20	10	2.2	4.50	120	115	1.04 ^#^
Male	23	9.5	2.0 ^††^	4.75 ^‡^	97	53 *	1.81
Female	23	9.3	1.5 ^†^	6.27 ^‡^	114	116	0.98 ^#^
Female	25	9.3	2.1	4.48	147 **	151 **	0.97 ^#^
Undernutrition							
Male	15	9.9	2.2	4.50	155 **	65 *	2.37
Female	15	10.6	1.9 ^††^	5.58 ^‡^	103	152 **	0.68 ^#^
Female	16	10.2	2.2	4.64	87	111	0.79 ^#^
Female	18	8.6	1.9 ^††^	4.68	95	132 **	0.72 ^#^
Female	31	9.7	1.7 ^†^	5.71 ^‡^	96	141 **	0.68 ^#^

Legend: BMI: body mass index. ^a^ Serum calcium (Ca) normal levels in children (8.8–10.8 mg/dL) and adults (9–10.5 mg/dL) [36]. ^b^ Serum magnesium (Mg): symptomatic hypomagnesemia (<1.22 mg/dL), asymptomatic hypomagnesemia (1.22–1.82 mg/dL), chronic latent magnesium deficiency (CLMD, 1.82–2.07 mg/dL), health (2.07–2.32 mg/dL), asymptomatic hypermagnesemia (2.32–4.86 mg/dL), symptomatic hypermagnesemia (>4.86 mg/dL) [37]. ^†^ CLMD, ^††^ asymptomatic hypomagnesemia. Serum Ca/Mg ratio (3.91–4.70) [26]. Ca/Mg intake ratio 1.70–2.60 [39]. ^‡^ High serum Ca/Mg ratio. Normal dietary Ca and Mg intake ranges from 80–120% Dietary Reference Intake (%DRI). * Low intake. ** High intake. ^#^ Low Ca/Mg intake ratio.

**Table 3:** Significant differences between cystic fibrosis patients (*n* = 17 *).

**Colonization**	**Yes**	**No**	
Magnesium intake (%DRI)	107 ± 35	155 ± 18	0.009 *
Calcium/magnesium intake ratio	1.31 ± 0.52	0.75 ± 0.13	0.008 *
**Nutritional Status**	**Undernutrition**	**Eutrophic**	
Iron intake (%DRI)	132 ± 20	252 ± 118	0.045 *
**Acute phase reactants**	**CRP high**	**Normal**	
Nitrogen balance	2.7 ± 4.3	13.9	0.034 *
**Respiratory function**	**Sufficient**	**Insufficient**	
Nitrogen intake	15.1 ± 2.7	20.6 ± 2.0	0.000 *
Nitrogen balance	0.7 ± 4.3	6.3 ± 4.9	0.034 *
**Pancreatic function**	**Sufficient**	**Insufficient**	
Nitrogen balance	8.9 ± 6.4	2.3 ± 4.3	0.048 *

Legend: %DRI: Percentage of Dietary Reference Intake. * *p* < 0.05.

**Table 4:** Regression analysis between serum level and dietary intake of magnesium (Mg) and calcium (Ca), serum and dietary Ca/Mg ratios, and nutritional parameters.

Serum Magnesium	Serum Calcium	Serum Ca/Mg Ratio	Magnesium Intake	Calcium Intake	Ca/Mg Intake Ratio
Linear	regression	analyses			
*r* = 0.234, *p* = 0.049 Age in years					
	*r* = 0.789, *p* = 0.003 kilogram’s fat mass by BIA		*r* = 0.444, *p* = 0.005 height-for-age Z-score	*r* = 0.418, *p* = 0.007 suprailiac skinfold Z-score	
	*r* = 0.332, *p* = 0.024 IGFBP3		*r* = 0.465, *p* = 0.004 Waterloo II	*r* = 0.262, *p* = 0.043 nutritional index	
			*r* = 0.530, *p* = 0.001 energy intake		*r* = 0.341, *p* = 0.017 vitamin C intake
			*r* = 0.3454, *p* = 0.004 protein intake		*r* = 0.449, *p* = 0.003 niacin intake
			*r* = 0.532, *p* = 0.001 total lipids intake		*r* = 0.309, *p* = 0.025 vitamin B12 intake
			*r* = 0.3261, *p* = 0.043 folic acid intake		
			*r* = 0.335, *p* = 0.019 zinc intake		
			*r* = 0.388, *p* = 0.017 Ca/Mg intake ratio		
	*r* = 0.557, *p* = 0.001 beta-carotene	*r* = 0.388, *p* = 0.017 serum vitamin B12	*r* = 0.359, *p* = 0.018 serum vitamin E		
*r* = 0.273, *p* = 0.038 serum phosphorus	*r* = 0.498, *p* = 0.002 serum total proteins			*r* = 0.394, *p* = 0.009 serum zinc	*r* = 0.327, *p* = 0.021 zinc/copper ratio
*r* = 0.807, *p* = 0.000 serum Ca/Mg ratio					*r* = 0.434, *p* = 0.006 HDL-cholesterol
*r* = 0.477, *p* = 0.003 creatinine		*r* = 0.429, *p* = 0.006 creatinine			
*r* = 0.293, *p* = 0.030 alkaline phosphatase					
	*r* = 0.240, *p* = 0.046 transferrin		*r* = 0.397, *p* = 0.009 transferrin saturation index	*r* = 0.345, *p* = 0.017 transferrin	*r* = 0.250, *p* = 0.049 transferrin saturation index
	*r* = 0.387, *p* = 0.013 urine nitrogen	*r* = 0.270, *p* = 0.047 urine creatinine			
		*r* = 0.298, *p* = 0.035 urine phosphorus			
*r* = 0.365, *p* = 0.017 basophiles	*r* = 0.281, *p* = 0.035 MCH				
*r* = 0.364, *p* = 0.013 lymphocytes CD8	*r* = 0.268, *p* = 0.040 platelets	*r* = 0.533, *p* = 0.001 IgM			
Multilinear	regression	analyses			
			*r* = 0.712, *p* = 0.000 Waterloo II, head circumference		*r* = 0.628, *p* = 0.002 niacin, folic acid intake
*r* = 0.907, *p* = 0.000 vitamin C, protein, iron, vitamin B12, polyunsaturated fat intake		*r* = 0.686, *p* = 0.002 energy, iron, vitamin C intake	*r* = 0.906, *p* = 0.000 total lipids, vitamin B12, calcium intake		*r* = 0.627, *p* = 0.002 vitamin B12, niacin intake
*r* = 0.590, *p* = 0.003 serum iron, alkaline phosphatase			*r* = 0.682, *p* = 0.001 total lipids, zinc intake	*r* = 0.639, *p* = 0.001 serum zinc and iron	
*r* = 0.986, *p* = 0.000 serum Ca/Mg ratio, calcium	*r* = 0.508, *p* = 0.010 eosinophiles, MCV	*r* = 0.985, *p* = 0.000 serum magnesium and calcium		*r* = 0.615, *p* = 0.003 platelet, eosinophiles	

Legend: BIA: bioelectrical impedance analysis; IGFBP3: insulin-like growth factor-binding protein 3; MCH: mean corpuscular hemoglobin; Ig: immunoglobulin; MCV: mean corpuscular volume.

## References

[1] Jaworska J., Marach-Mocarska A., Sands D. (2020). Uncommon clinical presentation of cystic fibrosis in a patient homozygous for a rare CFTR mutation: A case report. BMC Pediatr..

[2] Elborn J.S. (2016). Cystic fibrosis. Lancet.

[3] Davies J.C., Alton E.W.F.W., Bush A. (2007). Cystic fibrosis. Br. Med. J..

[4] Wang Y., Wrennall J.A., Cai Z., Li H., Sheppard D.N. (2014). Understanding how cystic fibrosis mutations disrupt CFTR function: From single molecules to animal models. Int. J. Biochem. Cell Biol..

[5] Culhane S., George C., Pearo B., Spoede E. (2013). Malnutrition in cystic fibrosis: A review. Nutr. Clin. Pract..

[6] Schrijver I. (2011). Mutation distribution in expanded screening for cystic fibrosis: Making up the balance in a context of ethnic diversity. Clin. Chem..

[7] Matel J.L., Milla C.E. (2009). Nutrition in cystic fibrosis. Semin. Respir. Crit. Care Med..

[8] Brownell J.N., Bashaw H., Stallings V.A. (2019). Growth and Nutrition in Cystic Fibrosis. Semin. Respir. Crit. Care Med..

[9] Turck D., Braegger C.P., Colombo C., Declercq D., Morton A., Pancheva R., Robberecht E., Stern M., Strandvik B., Wolfe S. (2016). ESPEN-ESPGHAN-ECFS guidelines on nutrition care for infants, children, and adults with cystic fibrosis. Clin. Nutr..

[10] Gupta A., Eastham K.M., Wrightson N., Spencer D.A. (2007). Hypomagnesemia in cystic fibrosis patients referred for lung transplant assessment. J. Cyst. Fibros..

[11] Gröber U., Schmidt J., Kisters K. (2015). Mg in prevention and therapy. Nutrients.

[12] Reddy S.T., Soman S.S., Yee J. (2018). Mg balance and measurement. Adv. Chronic Kidney Dis..

[13] Nielsen F.H., Johnson L.A.K. (2017). Data from controlled metabolic ward studies provide guidance for the determination of status indicators and dietary requirements for magnesium. Biol. Trace Elem. Res..

[14] Barbagallo M., Veronese N., Dominguez L.J. (2021). Magnesium in Aging, Health and Diseases. Nutrients.

[15] Wu J., Xun P., Tang Q., Cai W., He K. (2017). Circulating magnesium levels and incidence of coronary heart diseases, hyper-tension, and type 2 diabetes mellitus: A meta-analysis of prospective cohort studies. Nutr. J..

[16] Adebamowo S.N., Jimenez M.C., Chiuve S.E., Spiegelman D., Willett W.C., Rexrode K.M. (2014). Plasma magnesium and risk of ischemic stroke among women. Stroke.

[17] De Baaij J.H.F., Hoenderop J.G.J., Bindels R.J.M. (2015). Mg in Man: Implications for Health and Disease. Physiol. Rev..

[18] He K., Liu K., Daviglus M.L., Morris S.J., Loria C.M., Van Horn L., Jacobs D.R., Savage P.J. (2006). Magnesium intake and incidence of metabolic syndrome among young adults. Circulation.

[19] DiNicolantonio J.J., Liu J., O’Keefe J.H. (2018). Mg for the prevention and treatment of cardiovascular disease. Open Heart.

[20] Liguori I., Russo G., Curcio F., Bulli G., Aran L., Della-Morte D., Gargiulo G., Testa G., Cacciatore F., Bonaduce D. (2018). Oxidative stress, aging, and diseases. Clin. Interv. Aging.

[21] Ferre S., Li X., Adams-Huet B., Maalouf N.M., Sakhaee K., Toto R.D., Moe O.W., Neyra J.A. (2018). Association of serum magnesium with all-cause mortality in patients with and without chronic kidney disease in the Dallas Heart Study. Nephrol. Dial. Transpl..

[22] Hermes Sales C., Azevedo Nascimento D., Queiroz Medeiros A.C., Costa Lima K., Campos Pedrosa L.F., Colli C. (2014). There is chronic latent Mg deficiency in apparently healthy university students. Nutr. Hosp..

[23] Price C.T., Langford J.R., Liporace F.A. (2012). Essential Nutrients for Bone Health and a Review of their Availability in the Average North American Diet. Open Orthop. J..

[24] Lutsey P.L., Alonso A., Michos E.D., Loehr L.R., Astor B.C., Coresh J., Folsom A.R. (2014). Serum magnesium, phosphorus, and calcium are associated with risk of incident heart failure: The Atherosclerosis Risk in Communities (ARIC) Study. Am. J. Clin. Nutr..

[25] Sato H., Takeuchi Y., Matsuda K., Saito A., Kagaya S., Fukami H., Ojima Y., Nagasawa T. (2017). Evaluation of the Predictive Value of the Serum Calcium-Magnesium Ratio for All-Cause and Cardiovascular Mortality in Incident Dialysis Patients. Cardiorenal. Med..

[26] Li Q., Chen Q., Zhang H., Xu Z., Wang X., Pang J., Ma J., Ling W., Li D. (2020). Associations of serum magnesium levels and calcium-magnesium ratios with mortality in patients with coronary artery disease. Diabetes Metab..

[27] Escobedo Monge M.F., Barrado E., Alonso Vicente C., Redondo del Río M.P., Manuel Marugán de Miguelsanz J. (2019). Zinc Nutritional Status in Patients with Cystic Fibrosis. Nutrients.

[28] Escobedo-Monge M.F., Barrado E., Alonso Vicente C., Escobedo-Monge M.A., Torres-Hinojal M.C., Marugán-Miguelsanz J.M., Redondo del Río M.P. (2020). Copper and Copper/Zinc Ratio in a Series of Cystic Fibrosis Patients. Nutrients.

[29] Frisancho A.R. (1981). New norms of upper limb fat and muscle areas for assessment of nutritional status. Am. J. Clin. Nutr..

[30] Hernández M., Sobradillo B., Aguirre A., Aresti U., Bilbao A., Fernández-Ramos C., Lizárraga A., Lorenzo H., Madariaga L., Rica I. (1985). Curvas y Tablas de Crecimiento (Estudios Longitudinal y Transversal).

[31] Martínez M.J., Redondo D., Conde F., Redondo P., Franch M.A., de CyL J. (2009). Gráficas Longitudinales de Velocidad de Conducción Media de Ultrasonidos en Falanges. Estudio Nutricional de Castilla y León.

[32] Mataix Verdú J., García Diaz J. (2005). NUTRIBER. V. 1.0..

[33] Cuervo M., Corbalán M., Baladía E., Cabrerizo L., Formiguera X., Iglesias C., Lorenzo H., Polanco I., Quiles J., De Avila M.D.R. (2009). Comparison of dietary reference intakes (DRI) between different countries of the European Union, the United States and the World Health Organization. Nutr. Hosp..

[34] Jahnen-Dechent W., Ketteler M. (2012). Mg basics. Clin. Kidney J..

[35] Ahmed F., Mohammed A. (2019). Magnesium: The forgotten electrolyte—A review on hypomagnesemia. Med. Sci..

[36] Pagana K.D., Pagana T.J., Pagana T.N. (2019). Mosby’s Diagnostic & Laboratory Test Reference.

[37] Costello R.B., Elin R.J., Rosanoff A., Wallace T.C., Guerrero-Romero F., Hruby A., Lutsey P.L., Nielsen F.H., Rodriguez-Moran M., Song Y. (2016). Perspective: The Case for an Evidence-Based Reference Interval for Serum Magnesium: The Time Has Come12345. Adv. Nutr..

[38] Goltzman D., Feingold K.R., Anawalt B., Boyce A., Chrousos G., de Herder W.W., Dhatariya K., Dungan K., Hershman J.M., Hofland J., Kalra S. (2000). Hypercalcemia. Endotext [Internet].

[39] Costello R.B., Rosanoff A., Dai Q., Saldanha L.G., Potischman N.A. (2021). Perspective: Characterization of Dietary Supplements Containing Calcium and Magnesium and Their Respective Ratio—Is a Rising Ratio a Cause for Concern?. Adv. Nutr..

[40] Escobedo-Monge M.F., Torres-Hinojal M.C., Barrado E., Escobedo-Monge M.A., Marugán-Miguelsanz J.M. (2021). Zinc Nutritional Status in a Series of Children with Chronic Diseases: A Cross-Sectional Study. Nutrients.

[41] Escobedo-Monge M.F., Barrado E., Parodi-Román J., Escobedo-Monge M.A., Torres-Hinojal M.C., Marugán-Miguelsanz J.M. (2021). Copper and Copper/Zn Ratio in a Series of Children with Chronic Diseases: A Cross-Sectional Study. Nutrients.

[42] Workinger J.L., Doyle R.P., Bortz J. (2018). Challenges in the diagnosis of Mg status. Nutrients.

[43] DiNicolantonio J.J., O’Keefe J.H., Wilson W. (2018). Subclinical Mg deficiency: A principal driver of cardiovascular disease and a public health crisis. Open Heart Vol..

[44] Steele E.M., Popkin B.M., Swinburn B., Monteiro C.A. (2017). The share of ultra-processed foods and the overall nutritional quality of diets in the US: Evidence from a nationally representative cross-sectional study. Popul. Health Metr..

[45] Cascella M., Vaqar S. (2022). Hypermagnesemia. StatPearls [Internet].

[46] Razzaque M.S. (2018). Magnesium: Are We Consuming Enough?. Nutrients.

[47] Spatling L., Classen H.G., Kolpmann W.R., Manz F., Rob P.M., Schimatschek H.F., Vierling W., Vormann J., Weigert A., Wink K. (2000). Diagnostik des Magnesiummangels. Aktuelle Empfehlungen der Gesellschaft für MagnesiumForschung e. V. Fortschr. Med. Orig..

[48] Micke O., Vormann J., Kraus A., Kisters K. (2021). Serum magnesium: Time for a standardized and evidence-based reference range. Magnes. Res..

[49] Mason J.B., Goldman L., Schafer A.I. (2016). Vitamins, trace minerals, and other micronutrients. Goldman-Cecil Medicine.

[50] Santi M., Milani G.P., Simonetti G.D., Fossali E.F., Bianchetti M.G., Lava S.A.G. (2016). Mg in cystic fibrosis—Systematic review of the literature. Pediatr. Pulmonol..

[51] Khairi T., Amer S., Spitalewitz S., Alasadi L. (2014). Severe Symptomatic Hypermagnesemia Associated with Over-the-Counter Laxatives in a Patient with Renal Failure and Sigmoid Volvulus. Case Rep. Nephrol..

[52] Musso C.G. (2009). Magnesium metabolism in health and disease. Int. Urol. Nephrol..

[53] Adler A.I., Gunn E., Haworth C.S., Bilton D. (2007). Characteristics of adults with and without cystic fibrosis-related diabetes. Diabet. Med..

[54] Danziger J., William J.H., Scott D.J., Lee J., Lehman L.W., Mark R.G., Howell M.D., Celi L.A., Mukamal K.J. (2013). Proton-pump inhibitor use is associated with low serum Mg concentrations. Kidney Int. Int. Soc. Nephrol..

[55] van der Haak N., King S.J., Crowder T., Kench A., Painter C., Saxby N. (2020). Nutrition Guidelines for Cystic Fibrosis in Australia and New Zealand Authorship Group and Interdisciplinary Steering Committee. Highlights from the nutrition guidelines for cystic fibrosis in Australia and New Zealand. J. Cyst. Fibros..

[56] Maguire D., Ross D.P., Talwar D., Forrest E., Naz Abbasi H., Leach J.P., Woods M., Zhu L.Y., Dickson S., Kwok T. (2019). Low serum Mg and 1-year mortality in alcohol withdrawal syndrome. Eur. J. Clin. Investig..

[57] Ayuk J., Gittoes N.J. (2014). Contemporary view of the clinical relevance of Magnesium homeostasis. Ann. Clin. Biochem..

[58] Swaminathan R. (2003). Mg metabolism and its disorders. Clin. Biochem. Rev..

[59] Hansen B.A., Bruserud Ø. (2018). Hypomagnesemia in critically ill patients. J. Intensive Care.

[60] Tarleton E.K. (2018). Factors influencing Mg consumption among adults in the United States. Nutr. Rev..

[61] Olza J., Aranceta-Bartrina J., González-Gross M., Ortega R., Serra-Majem L., Varela-Moreiras G., Gil Á. (2017). Reported Dietary Intake, Disparity between the Reported Consumption and the Level Needed for Adequacy and Food Sources of Calcium, Phosphorus, Mg and Vitamin D in the Spanish Population: Findings from the ANIBES Study. Nutrients.

[62] Wang Y., Wei J., Zeng C., Yang T., Li H., Cui Y., Xie D., Xu B., Liu Z., Li J. (2018). Association between serum Mg concentration and metabolic syndrome, diabetes, hypertension and hyperuricaemia in knee osteoarthritis: A cross-sectional study in Hunan Province, China. BMJ Open.

[63] Berg K.H., Ryom L., Faurholt-Jepsen D., Pressler T., Katzenstein T.L. (2017). Prevalence and characteristics of chronic kidney disease among Danish adults with cystic fibrosis. J. Cyst. Fibros..

[64] Moheet A., Moran A. (2017). CF-related diabetes: Containing the metabolic miscreant of cystic fibrosis. Pediatr. Pulmonol..

[65] Kastner-Cole D., Palmer C.N., Ogston S.A., Mehta A., Mukhopadhyay S. (2005). Overweight and obesity in deltaF508 homozygous cystic fibrosis. J. Pediatr..

[66] Litvin M., Yoon J.C., Leey Casella J., Blackman S.M., Brennan A.L. (2019). Energy balance and obesity in individuals with cystic fibrosis. J. Cyst. Fibros..

[67] Cystic Fibrosis. https://www.cysticfibrosis.org.uk.

[68] Toh J.W., Ong E., Wilson R. (2015). Hypomagnesaemia associated with long-term use of proton pump inhibitors. Gastroenterol. Rep..

[69] Laecke S.V., Biesen W.V., Vanholder R. (2012). Hypomagnesaemia, the kidney and the vessels. Nephrol. Dial. Transpl..

[70] Li L., Streja E., Rhee C.M., Mehrotra R., Soohoo M., Brunelli S.M., Kovesdy C.P., Kalantar-Zadeh K. (2015). Hypomagnesemia and mortality in incident hemodialysis patients. Am. J. Kidney Dis..

[71] Masakane I., Nakai S., Ogata S., Kimata N., Hanafusa N., Hamano T., Wakai K., Wada A., Nitta K. (2012). Overview of regular dialysis treatment in Japan (as of 31 December 2009). Ther. Apher. Dial..

[72] Lee M.J., Alvarez J.A., Smith E.M., Killilea D.W., Chmiel J.F., Joseph P.M., Grossmann R.E., Gaggar A., Ziegler T.R., Tangpricha V. (2015). Vitamin D for Enhancing the Immune System in Cystic Fibrosis Investigators. Changes in Mineral Micronutrient Status During and After. Nutr. Clin. Pract..

[73] Bozoky Z., Ahmadi S., Milman T., Kim T.H., Du K., Di Paola M., Pasyk S., Pekhletski R., Keller J.P., Bear C.E. (2017). Synergy of cAMP and calcium signaling pathways in CFTR regulation. Proc. Natl. Acad. Sci. USA.

[74] Rimessi A., Vitto V., Patergnani S., Pinton P. (2021). Update on Calcium Signaling in Cystic Fibrosis Lung Disease. Front. Pharmacol..

[75] Antigny F., Norez C., Becq F., Vandebrouck C. (2011). CFTR and Ca signaling in cystic fibrosis. Front. Pharmacol..

[76] Aspray T.J., Collins J.F. (2017). Calcium: Basic Nutritional Aspects. Molecular, Genetic, and Nutritional Aspects of Major and Trace Minerals.

[77] Arnaud M.J. (2008). Update on the assessment of Mg status. Br. J. Nutr..

[78] Saris N.E.L., Mervaala E., Karppanen H., Khawaja J.Á., Lewenstam A. (2000). Mg^2+^: An update on physiological, clinical and analytical aspects. Clin. Chim. Acta.

[79] Kisters K., Wessels F., Küper H., Tokmak F., Krefting E.R., Gremmler B., Kosch M., Barenbrock M., Hausberg M. (2004). Increased calcium and decreased magnesium concentrations and an increased calcium/magnesium ratio in spontaneously hypertensive rats versus Wistar-Kyoto rats: Relation to arteriosclerosis. Am. J. Hypertens..

[80] Zhang H., Cao Y., Song P., Man Q., Mao D., Hu Y., Yang L. (2021). Suggested Reference Ranges of Blood Mg and Ca Level in Childbearing Women of China: Analysis of China Adult Chronic Disease and Nutrition Surveillance (2015). Nutrients.

[81] Yuan Z., Liu C., Tian Y., Zhang X., Ye H., Jin L., Ruan L., Sun Z., Zhu Y. (2016). Higher levels of magnesium and lower levels of calcium in whole blood are positively correlated with the metabolic syndrome in a Chinese population: A Case-Control Study. Ann. Nutr. Metab..

[82] Office of Dietary Supplements: National Institutes of Health (2018). Magnesium. http://ods.od.nih.gov/factsheets/folate.

[83] Moore-Schiltz L., Albert J.M., Singer M.E., Swain J., Nock N.L. (2015). Dietary intake of calcium and Magnesium and the metabolic syndrome in the National Health and Nutrition Examination (NHANES) 2001–2010 data. Br. J. Nutr..

[84] Kousa A., Havulinna A.S., Moltchanova E., Taskinen O., Nikkarinen M., Eriksson J., Karvonen M. (2006). Calcium: Magnesium ratio in local groundwater and incidence of acute myocardial infarction among males in rural Finland. Environ. Health Perspect..

[85] Celik M., Koklu M., Gusoy E., Gungo M., Yasar S., Gormel S., Yildirim E., Gokoglan Y., Yuksel U.C., Kaul H.K. (2016). The serum calcium to Magnesium ratio in patients with acute coronary syndrome. Acta Med. Mediterr..

[86] Dai Q., Sandler R., Barry E., Summers R., Grau M., Baron J. (2012). Calcium, magnesium, and colorectal cancer. Epidemiology.

[87] Steck S.E., Omofuma O.O., Su L.J., Maise A.A., Woloszynska-Read A., Johnson C.S., Zhang H., Bensen J.T., Fontham E.T., Mohler J.L. (2018). Calcium, magnesium, and whole-milk intakes and high-aggressive prostate cancer in the North Carolina-Louisiana Prostate Cancer Project (PCaP). Am. J. Clin. Nutr..

[88] Shah S.C., Dai Q., Zhu X., Peek R.M., Roumie C., Shrubsole M.J. (2020). Associations between calcium and Magnesium intake and the risk of incident oesophageal cancer: An analysis of the NIH-AARP Diet and Health Study prospective cohort. Br. J. Cancer.

[89] Vázquez-Lorente H., Herrera-Quintana L., Molina-López J., Gamarra-Morales Y., López-González B., Miralles-Adell C., Planells E. (2020). Response of vitamin D after Magnesium intervention in a postmenopausal population from the province of Granada, Spain. Nutrients.

[90] Dai Q., Shrubsole M.J., Ness R.M., Schlundt D., Cai Q., Smalley W.E., Li M., Shyr Y., Zheng W. (2007). The relation of Magnesium and calcium intakes and a genetic polymorphism in the Magnesium transporter to colorectal neoplasia risk. Am. J. Clin. Nutr..

[91] Dai Q., Cantwell M.M., Murray L.J., Zheng W., Anderson L.A., Coleman H.G. (2016). Dietary magnesium, calcium:magnesium ratio and risk of reflux oesophagitis, Barrett’s oesophagus and oesophageal adenocarcinoma: A population-based case-control study. Br. J. Nutr..

[92] Dai Q., Shu X.O., Deng X., Xiang Y.B., Li H., Yang G., Shrubsole M.J., Ji B., Cai H., Chow W.H. (2013). Modifying effect of calcium/magnesium intake ratio and mortality: A population-based cohort study. BMJ Open.

[93] Zhao J., Giri A., Zhu X., Shrubsole M.J., Jiang Y., Guo X., Ness R., Seidner D.L., Giovannucci E., Edwards T.L. (2019). Calcium: Magnesium intake ratio and colorectal carcinogenesis, results from the Prostate, Lung, Colorectal, and Ovarian cancer screening trial. Br. J. Cancer.

[94] Hibler E.A., Zhu X., Shrubsole M.J., Hou L., Dai Q. (2020). Physical activity, dietary calcium to Magnesium intake and mortality in the National Health and Examination Survey 1999–2006 cohort. Int. J. Cancer.

[95] Zhu X., Borenstein A.R., Zheng Y., Zhang W., Seidner D.L., Ness R., Murff H.J., Li B., Shrubsole M.J., Yu C. (2020). Ca:Mg ratio, APOE cytosine modifications, and cognitive function: Results from a randomized trial. J. Alzheimer’s Dis..

[96] Lu L., Chen C., Yang K., Zhu J., Xun P., Shikany J.M., He K. (2020). Mg intake is inversely associated with risk of obesity in a 30-year prospective follow-up study among American young adults. Eur. J. Nutr..

[97] Tham A., Katz T.E., Sutherland R.E., Garg M., Liu V., Tong C.W., Brunner R., Quintano J., Collins C., Ooi C.Y. (2020). Micronutrient intake in children with cystic fibrosis in Sydney, Australia. J. Cyst. Fibros..

[98] Blache D., Devaux S., Joubert O., Loreau N., Schneider M., Durand P., Prost M., Gaume V., Adrian M., Laurant P. (2006). Long-term moderate magnesium-deficient diet shows relationships between blood pressure, inflammation and oxidant stress defense in aging rats. Free Radic. Biol. Med..

[99] Micke O., Vormann J., Kisters K. (2020). Mg and COVID-19—Some Further Comments—A Commentary on Wallace TC. Combating COVID-19 and Building Immune Resilience: A Potential Role for Mg Nutrition?. J. Am. Coll. Nutr..

[100] Schwalfenberg G.K., Genuis S.J. (2017). The Importance of Mg in Clinical Healthcare. Scientifica.

[101] Gontijo-Amaral C., Guimarães E.V., Camargos P. (2012). Oral Mg supplementation in children with cystic fibrosis improves clinical and functional variables: A double-blind, randomized, placebo-controlled crossover trial. Am. J. Clin. Nutr..

[102] Hassan S.A., Ahmed I., Nasrullah A., Haq S., Ghazanfar H., Sheikh A.B., Zafar R., Askar G., Hamid Z., Khushdil A. (2017). Comparison of Serum Mg Levels in Overweight and Obese Children and Normal Weight Children. Cureus.

[103] Castellani C., Duff A.J.A., Bell S.C., Heijerman H.G., Munck A., Ratjen F., Sermet-Gaudelus I., Southern K.W., Barben J., Flume P.A. (2018). ECFS best practice guidelines: The 2018 revision. J. Cyst. Fibros..

[104] Kilinc A.A., Beser O.F., Ugur E.P., Cokugras F.C., Cokugras H. (2021). The effects of nutritional status and intervention on pulmonary functions in pediatric cystic fibrosis patients. Pediatr. Int..

[105] Nielsen F.H. (2014). Effects of Magnesium depletion on inflammation in chronic disease. Curr. Opin. Clin. Nutr. Metab. Care.

[106] Institute of Medicine (US) Standing Committee on the Scientific Evaluation of Dietary Reference Intakes (1997). Dietary Reference Intakes for Calcium, Phosphorus, Mg, Vitamin D., and Fluoride.

[107] Das S., Sanchez J.J., Alam A., Haque A., Mahfuz M., Ahmed T., Long K.Z. (2020). Dietary Magnesium, Vitamin D, and Animal Protein Intake and Their Association to the Linear Growth Trajectory of Children from Birth to 24 Months of Age: Results from MAL-ED Birth Cohort Study Conducted in Dhaka, Bangladesh. Food Nutr. Bull..

[108] de Lamas C., de Castro M.J., Gil-Campos M., Gil Á., Couce M.L., Leis R. (2019). Effects of Dairy Product Consumption on Height and Bone Mineral Content in Children: A Systematic Review of Controlled Trials. Adv. Nutr..

[109] Moussa Z., Judeh Z.M., Ahmed S.A., Das K., Das S., Biradar M.S., Bobbarala V., Tata S.S. (2017). Nonenzymatic Exogenous and Endogenous Antioxidants. Free Radical Medicine and Biology.

[110] Cho S., Chae J.S., Shin H., Shin Y., Song H., Kim Y., Yoo B.C., Roh K., Cho S., Kil E.-J. (2018). Hormetic dose response to L-ascorbic acid as an anti-cancer drug in colorectal cancer cell lines according to SVCT-2 expression. Sci. Rep..

[111] Cho S., Chae J.S., Shin H., Shin Y., Kim Y., Kil E.J., Byun H.S., Cho S.H., Park S., Lee S. (2020). Enhanced Anticancer Effect of Adding Mg to Vitamin C Therapy: Inhibition of Hormetic Response by SVCT-2 Activation. Transl. Oncol..

[112] Tan C.W., Ho L.P., Kalimuddin S., Cherng B.P.Z., Teh Y.E., Thien S.Y., Wong H.M., Tern P.J.W., Chandran M., Chay J.W.M. (2020). Cohort study to evaluate the effect of vitamin D, magnesium, and vitamin B12 in combination on progression to severe outcomes in older patients with coronavirus (COVID-19). Nutrition.

[113] Shi Z.M., Hu X.S., Yuan B.J., Gibson R., Dai Y., Garg M. (2008). Association between magnesium: Iron intake ratio and diabetes in Chinese adults in Jiangsu Province. Diabet. Med..

[114] Sampaio F.A., Feitosa M.M., Sales C.H., Costa D.M., Cruz K.J.C., Oliveira F.E., Colli C., do Nascimento Marreiro D. (2014). Influence of Mg on biochemical parameters of iron and oxidative stress in patients with type 2 diabetes. Nutr. Hosp..

[115] Polzikov M., Blinov D., Barakhoeva Z., Vovk L., Fetisova Y., Ovchinnikova M., Tischenko M., Zorina I., Yurasov V., Ushakova T. (2022). Association of the Serum Folate and Total Calcium and Mg Levels Before Ovarian Stimulation with Outcomes of Fresh In Vitro Fertilization Cycles in Normogonadotropic Women. Front. Endocrinol..

[116] Guo W., Nazim H., Liang Z., Yang D. (2016). Mg deficiency in plants: An urgent problem. Crop J..

[117] Quinn S.J., Thomsen A.R., Egbuna O., Pang J., Baxi K., Goltzman D., Pollak M., Brown E.M. (2013). CaSR-mediated interactions between calcium and Mg homeostasis in mice. Am. J. Physiol. Endocrinol. Metab..

[118] El-shehawy E.L., Allah S.B., Mahmoud A.T., Mansour A.E., Elsayed O.M. (2020). The Impact of Serum Mg Level Disorders on Parathyroid Hormone and Alkaline Phosphatase Levels in Patients with Chronic Kidney Disease Stage 5 under Maintenance Hemodialysis. Benha J. Appl. Sci..

[119] Nielsen F.H. (2008). Marginal Zinc Deficiency Increases Mg Retention and Impairs Calcium Utilization in Rats. Biol. Trace Elem. Res..

[120] O’Connor J.P., Kanjilal D., Teitelbaum M., Lin S.S., Cottrell J.A. (2020). Zinc as a Therapeutic Agent in Bone Regeneration. Materials.

[121] Hamedifard Z., Farrokhian A., Reiner Ž., Bahmani F., Asemi Z., Ghotbi M., Taghizadeh M. (2020). The effects of combined Mg and zinc supplementation on metabolic status in patients with type 2 diabetes mellitus and coronary heart disease. Lipids Health Dis..

[122] Hamasaki H., Kawashima Y., Yanai H. (2016). Serum Zn/Cu Ratio Is Associated with Renal Function, Glycemic Control, and Metabolic Parameters in Japanese Patients with and without Type 2 Diabetes: A Cross-sectional Study. Front. Endocrinol..

[123] Granados A., Chan C.L., Ode K.L., Moheet A., Moran A., Holl R. (2019). Cystic fibrosis related diabetes: Pathophysiology, screening and diagnosis. J. Cyst. Fibros..

[124] Găman M.A., Dobrică E.C., Cozma M.A., Antonie N.I., Stănescu A.M., Găman A.M., Diaconu C.C. (2021). Crosstalk of Mg and Serum Lipids in Dyslipidemia and Associated Disorders: A Systematic Review. Nutrients.

[125] Ohira T., Peacock J.M., Iso H., Chambless L.E., Rosamond W.D., Folsom A.R. (2009). Serum and dietary Mg and risk of ischemic stroke: The Atherosclerosis Risk in Communities Study. Am. J. Epidemiol..

[126] Huang J.H., Lu Y.F., Cheng F.C., Lee J.N., Tsai L.C. (2012). Correlation of Mg intake with metabolic parameters, depression and physical activity in elderly type 2 diabetes patients: A cross-sectional study. Nutr. J..

[127] Maktabi M., Jamilian M., Amirani E., Chamani M., Asemi Z. (2018). The effects of Mg and vitamin E co-supplementation on parameters of glucose homeostasis and lipid profiles in patients with gestational diabetes. Lipids Health Dis..

[128] Afzali H., Jafari Kashi A.H., Momen-Heravi M., Razzaghi R., Amirani E., Bahmani F., Gilasi H.R., Asemi Z. (2019). The effects of Mg and vitamin E co-supplementation on wound healing and metabolic status in patients with diabetic foot ulcer: A randomized, double-blind, placebo-controlled trial. Wound Repair. Regen..

[129] Singh R.B., Rastogi S.S., Mani U.V., Seth J., Devi L. (1991). Does dietary Mg modulate blood lipids?. Biol. Trace Elem. Res..

[130] Ogun A.S., Adeyinka A. (2022). Biochemistry, Transferrin. StatPearls [Internet].

[131] Ścibior A., Hus I., Mańko J., Jawniak D. (2020). Evaluation of the level of selected iron-related proteins/receptors in the liver of rats during separate/combined vanadium and Mg administration. J. Trace Elem. Med. Biol..

[132] Sabbir M.G. (2020). CAMKK2-CAMK4 signaling regulates transferrin trafficking, turnover, and iron homeostasis. Cell Commun. Signal..

[133] El Mallah C., Ghattas H., Shatila D., Francis S., Merhi K., Hlais S., Toufeili I., Obeid O. (2016). Urinary Mg, Calcium, and Phosphorus to Creatinine Ratios of Healthy Elementary School Lebanese Children. Biol. Trace Elem. Res..

[134] Campuzano S., Díaz J.J., Bousoño C., Rodríguez M., Campos C., Málaga S. (2009). Riesgo de urolitiasis en pacientes con fibrosis quística [Risk of urolithiasis in patients with cystic fibrosis]. Nefrología.

[135] Tam M., Gómez S., González-Gross M., Marcos A. (2003). Possible roles of Mg on immune system. Eur. J. Clin. Nutr..

[136] Sugimoto J., Romani A.M., Valentin-Torres A.M., Luciano A.A., Ramirez Kitchen C.M., Funderburg N., Mesiano S., Bernstein H.B. (2012). Mg decreases inflammatory cytokine production: A novel innate immunomodulatory mechanism. J. Immunol..

[137] Feske S., Skolnik E.Y., Prakriya M. (2012). Ion channels and transporters in lymphocyte function and immunity. Nat. Rev. Immunol..

[138] Nielsen F.H. (2018). Mg deficiency and increased inflammation: Current perspectives. J. Inflamm. Res..

[139] Maier J.A., Castiglioni S., Locatelli L., Zocchi M., Mazur A. (2020). Mg and inflammation: Advances and perspectives. Semin. Cell Dev. Biol..

[140] Piuri G., Zocchi M., Della Porta M., Ficara V., Manoni M., Zuccotti G.V., Pinotti L., Maier J.A., Cazzola R. (2021). Mg in Obesity, Metabolic Syndrome, and Type 2 Diabetes. Nutrients.

[141] Yamanaka R., Tabata S., Shindo Y., Hotta K., Suzuki K., Soga T., Oka K. (2016). Mitochondrial Mg^2+^ homeostasis decides cellular energy metabolism and vulnerability to stress. Sci. Rep..

[142] Romani A., Scarpa A. (1992). Regulation of cell Mg^2+^. Arch. Biochem. Biophys..

[143] Aleksandrov A.A., Riordan J.R. (1998). Regulation of CFTR ion channel gating by MgATP. FEBS Lett..

[144] Grebert C., Becq F., Vandebrouck C. (2019). Focus on TRP channels in cystic fibrosis. Cell Calcium.

[145] Scanlan B.J., Tuft B., Elfrey J.E., Smith A., Zhao A., Morimoto M., Chmielinska J.J., Tejero-Taldo M.I., Mak I.T., Weglicki W.B. (2007). Intestinal inflammation caused by magnesium deficiency alters basal and oxidative stress-induced intestinal function. Mol. Cell Biochem..

[146] Sanders N.N., De Smedt S.C., Demeester J., Acoxyribonucle I., Mcgrath B.M., Walsh G. (2006). Directory of Therapeutic Enzymes.

[147] Mathew A.A., Panonnummal R. (2021). ‘Magnesium’—The master cation—As a drug—Possibilities and evidences. Biometals.

